# Whey Protein Hydrolysate Ameliorated High-Fat-Diet Induced Bone Loss via Suppressing Oxidative Stress and Regulating GSK-3β/Nrf2 Signaling Pathway

**DOI:** 10.3390/nu15132863

**Published:** 2023-06-24

**Authors:** Tingting Bu, Ju Huang, Yue Yu, Peilong Sun, Kai Yang

**Affiliations:** Department of Food Science and Technology, Zhejiang University of Technology, Hangzhou 310014, China; butingting@zjut.edu.cn (T.B.); h185360@163.com (J.H.); 221122260071@zjut.edu.cn (Y.Y.); sun_pl@zjut.edu.cn (P.S.)

**Keywords:** whey protein hydrolysate, high-fat diet, obesity, osteoporosis, oxidative stress, runt-related transcription factor 2, glycogen synthase kinase-3β, nuclear factor-erythroid 2-related factor 2

## Abstract

Long-term hypercaloric intake such as a high-fat diet (HFD) could act as negative regulators on bone remodeling, thereby inducing bone loss and bone microarchitecture destruction. Currently, food-derived natural compounds represent a promising strategy to attenuate HFD-induced bone loss. We previously prepared a whey protein hydrolysate (WPH) with osteogenic capacity. In this study, we continuously isolated and identified an osteogenic and antioxidant octapeptide TPEVDDA from WPH, which significantly promoted the alkaline phosphatase activities on MC3T3-E1 cells and exerted DPPH radical scavenging capacity. We then established an HFD-fed obese mice model with significantly imbalanced redox status and reduced bone mass and further evaluated the effects of different doses of WPH on ameliorating the HFD-induced bone loss and oxidative damages. Results showed that the administration of 2% and 4% WPH for 12 weeks significantly restored perirenal fat mass, improved serum lipid levels, reduced oxidative stress, and promoted the activity of antioxidant enzymes; meanwhile, WPH significantly preserved bone mass and bone mechanical properties, attenuated the degradation of trabecular microstructure, and regulated serum bone metabolism biomarkers. The protein levels of Runx2, Nrf2, and HO-1, as well as the phosphorylation level of GSK-3β in tibias, were notably activated by WPH. Overall, we found that the potential mechanism of WPH on ameliorating the HFD-induced bone loss mainly through its antioxidant and osteogenic capacity by activating Runx2 and GSK-3β/Nrf2 signaling pathway, demonstrating the potential of WPH to be used as a nutritional strategy for obesity and osteoporosis.

## 1. Introduction

The global prevalence of obesity has increased considerably over the past decades, which can be attributed to excessive energy consumption such as high-fat diets (HFD) [1]. Obesity has also been implicated as a potential precursor of many cardiovascular diseases, type 2 diabetes, and osteoporosis [2]. Noteworthy, accumulating epidemiological investigations pointed out that overweight and obese individuals tended to have decreased bone mass and compromised mechanical properties [3,4]. Scientific evidence revealed that long-term intake of HFD induced high levels of reactive oxygen species (ROS), which triggered oxidative stress and weakened the antioxidant defense system [5,6]. The increased oxidative damage not only shifted bone remodeling towards a negative balance between bone formation and resorption [7], but also promoted the production of proinflammatory cytokines such as tumor necrosis factor α (TNF–α), interleukin 1 (IL-1), and IL-6, which could disturb the coordinated osteoprotegerin (OPG)/receptor activator of nuclear factor kappa B ligand (RANKL)/RANK axis [8,9].

The nuclear factor-erythroid 2-related factor 2 (Nrf2) is the master regulator of redox hemostasis, which can resist oxidative damage by activating downstream antioxidant proteins such as heme oxygenase 1 (HO-1) and promoting the antioxidant enzymes such as catalase (CAT), superoxide dismutase (SOD), and glutathione peroxidase (GSH-Px) [10,11]. Studies have reported that the activation of Nrf2 could protect against osteoporosis by inhibiting oxidative damage [12]. Meanwhile, the Wnt/glycogen synthase kinase-3β (GSK-3β) signaling is the key pathway regulating bone formation [13], and studies have found that the phosphorylation GSK-3β can increase the transcription of Nrf2 [14]. Therefore, the regulation of Wnt/GSK-3β signaling might be a promising way for osteoporosis caused by obesity.

There has been a growing interest in preventing and ameliorating obesity and its related comorbidities by using food-derived natural compounds [15], such as polyphenols [16], anthocyanins [17], polysaccharides [18], unsaturated fatty acids [19], and proteins/peptides [20,21]. Among these, food-derived bioactive peptides have attracted much attention owing to their diverse biological properties and high safety [22]. Whey protein is a good resource to prepare bioactive peptides as it possesses numerous proteins with biological activities such as β–lactoglobulin, α–lactalbumin, immunoglobulins, and lactoferrin [23]. Whey-derived antioxidant peptide YVEEL ameliorated osteoporosis in an ovariectomized rat model by suppressing inflammation and enhancing osteogenesis [24]. We previously reported a whey protein enzymatic hydrolysate (WPH) exerted effects on activating osteogenesis and preventing ovariectomy-induced osteoporosis through regulating the p38/mitogen-activated protein kinase (MAPK)/Runx2 signaling pathway [25,26,27]. Although the anti-obesity/adipogenesis function of milk proteins, as well as their derived bioactive peptides, has been reported previously [28,29], the regulation effects of WPH on HFD-induced bone loss through redox modulation have not been sufficiently investigated. Thus, in this study, we first identified the peptides with dual capacities, antioxidant and osteogenic activities from WPH; then we established the HFD-induced osteoporotic mice model to examine the efficiency of WPH in ameliorating the HFD-induced bone loss, and revealed the inner mechanism from oxidative stress, and the Wnt/GSK-3β signaling.

## 2. Materials and Methods

### 2.1. Materials and Chemicals

Whey protein concentrate (WPC) powder (81.15 ± 0.34 g protein per 100 g powder) was purchased from Fonterra Co., Ltd. (Auckland, New Zealand). Proteases were provided by Amano Enzyme Inc. (Nagoya, Japan). The octapeptide Thr-Pro-Glu-Val-Asp-Asp-Ala (TPEVDDA) was chemically synthesized by China Peptide Company (Shanghai, China) and the purity was above 98%. All other chemicals were purchased from Sinopharm Chemical Reagent Co. (Shanghai, China).

### 2.2. Preparation of Whey Protein Hydrolysate (WPH)

The WPH was prepared according to our previously reported method [26]. Briefly, 5 g WPC powder was dissolved in 100 mL pure water and was sequentially hydrolyzed by 1% (*w*/*w*) THERMOASE C100 (65 °C, pH 7.5) and 1% Protease A “Amano” 2 SD (55 °C, pH 7.0) for 3 h, respectively. The hydrolysis was terminated by boiling and the hydrolysate was centrifugated at 10,000× *g* for 10 min. The supernatant was further purified through a D101 macroporous resin column to separate the whey protein hydrolysate (WPH). The lyophilized WPH was used for further studies.

### 2.3. Characterization of the Peptide Sequences

The separation of WPH was conducted on a semi-preparative RP-HPLC (e2695, Waters, Milford, MA, USA) equipped with a C18 column (10 mm × 250 mm, 5 μm), with a liner gradient (3–40% in 40 min) of acetonitrile (containing 0.1% trifluoroacetic acid) at a flow rate of 1 mL/min. The individually gathered fractions were lyophilized and further dissolved in a culture medium (500 μg/mL) or distilled water (100 μg/mL), to evaluate the osteogenic and antioxidant activities, respectively, on the basis of activating the expression of alkaline phosphatase (ALP) in MC3T3-E1 osteoblast cells and the radical scavenging capacity on 1,1-diphenyl-2-picrylhydrazyl (DPPH). The most effective fraction was subjected to nano-HPLC-Orbitrap-ESI-MS (Thermo Scientific, Bremen, Germany), according to our previous method [30], to identify the peptide sequences.

### 2.4. Alkaline Phosphatase (ALP) Activity Assay

The MC3T3-E1 preosteoblast cells (subclone 4, CRL-2593), bought from ATCC (Manassas, VA, USA) were seeded on 6-well plates at a density of 1 × 10^5^ cells per well and were incubated in growth medium (α–MEM medium supplemented with 10% FBS and 1% penicillin/streptomycin, Hyclone/Thermo Fisher, Waltham, MA, USA). After the cells reached ~80% confluency (about 48 h), 0, 5, 10, 20, 40, and 80 μM whey peptide TPEVDDEA were, respectively, added into the growth medium and co-cultured with cells for 72 h to induce differentiation. Then, radioimmunoprecipitation assay (RIPA) lysis buffer (P0013B, Beyotime, Nantong, China) was added to each well to collect cell lysates. The ALP activity, which is the differentiation marker of osteoblasts, was evaluated by an ALP assay kit (Beyotime) and was represented as the unit activity per mg of BCA protein content.

### 2.5. Capacity of Scavenging DPPH Radicals

The scavenging potential of DPPH radicals was determined by the method of Vinholes et al. (2011) [31]. Octapeptide TPEVDDEA was dissolved in pure water to obtain the final concentration of 0, 5, 10, 20, 40, and 80 μM. A 50 μL sample of each concentration was added to a 96-well microplate as well as 150 μL of 0.2 mM DPPH solution. The plates were incubated for 30 min in the dark, then the absorbances were measured at a wavelength of 515 nm.

### 2.6. Animals and Treatment

All animal experimental procedures were approved by the Institutional Animal Care and Use Committee of Zhejiang Chinese Medical University (approval No. IACUC-12395). Four-week-old male C57BL/6J mice were purchased and housed in a pathogen-free facility where maintained a constant temperature (25 ± 2 °C) and a 12 h light/dark cycle. After acclimatization for 1 week, all mice were randomly assigned into 2 groups (4 mice/cage) and fed either with a normal diet (ND, 15% of total calories from fat, *n* = 16) or with a high-fat diet (HFD, 45% of total calories from fat, *n* = 48). After feeding for 8 weeks, eight mice in every two groups were randomly selected to verify the effect of HFD on bone status. The remaining HFD mice (*n* = 40) were further randomly divided into 5 groups (*n* = 8 for each group) to determine the effect of WPH on bones, i.e., HFD, HFD + 1% WPH (10 g/kg HFD, HFD-WPHL); HFD + 2% WPH (20 g/kg HFD, HFD-WPHM); HFD + 4% WPH (40 g/kg HFD, HFD-WPHH). Each mouse was numbered and weighed every 2 weeks, and all mice had ad libitum access to food and water throughout the duration of 12 weeks. The composition of the diets was listed in Table 1.

### 2.7. Sample Collection

At the end of 12 weeks of treatments, all mice were sacrificed after overnight fasting. The whole blood was carefully collected from the orbital sinus and was centrifuged (5000× *g*, 10 min, 4 °C) to collect supernatant serum. The liver and fat pads were excised and weighed. One side of the femur and tibia was separated and stored in formalin for analysis; the other side of the femur and tibia was removed and immediately stored in liquid nitrogen.

### 2.8. Serum Lipid and Glucose Status

Serum glucose, triacylglycerol, and total cholesterol were measured by diagnostic kits (Nanjing Jiancheng Biological Co., Nanjing, Jiangsu, China) according to the manufacturer’s instructions.

### 2.9. Mechanical Testing and Bone Composition

The mechanical properties of the femur were determined by the bending test using a texture analyzer, according to our previous study. The femur was parallel positioned onto the platform and the loading force was 1 N with a speed of 0.1 mm/s, during which the maximal loads and the ultimate loads were recorded. Then, the used femurs were dried in an oven at 110 °C for 24 h to record dry weight and further ashed in a muffle at 550 °C for 48 h to evaluate the contents of calcium and phosphorus after extracting with 1 M HCl, using a ZA3000 polarized Zeeman atomic absorption spectrophotometer (Hitachi Ltd., Tokyo, Japan).

### 2.10. Micro-CT Analysis

The Skycan 1176 μCT scanner (Bruker, Kontich, Belgium) was used to image the microstructures of distal femurs, as described previously. Right femurs were aligned perpendicularly and scanned at a resolution of 9 μm voxel to reconstruct the whole 3D images, by using the equipped NRecon software. Then, the trabecular bone was separated using the MicroView software (GE, Madison, WI, USA). The trabecular microarchitectures were characterized by determining the volumetric bone mineral density (vBMD), bone volume density (BV/TV), trabecular number (Tb.N), trabecular thickness (Tb.Th), and trabecular separation (Tb.Sp).

### 2.11. H&E Staining

Tibias were firstly fixed in 4% paraformaldehyde for 2 days, then were decalcified in 10% EDTA (W/V, pH 7.0) for 1 month. After decalcification, tibias were embedded in paraffin and cut into slices with 4 μm thickness. Hemotoxylin and eosin (H&E) staining was carried out on the slices, which were further sealed in neutral gum, observed under a microscope (Eclipse 400, Nikon, Tokyo, Japan), and calculated for trabecular area ratio.

### 2.12. Bone Metabolism Biomarkers and Antioxidant Enzymes in Serum

Antioxidant enzymes (SOD, GSH-PX, and CAT), malondialdehyde (MDA), bone formation biomarkers (osteocalcin (OCN) and OPG), and bone resorption biomarkers (tartrate-resistant acid phosphatase 5b (TRACP5b) and RANKL) in serum were assessed by corresponding enzyme-linked immunosorbent assay (ELISA) kits, purchased from Meibiao Biological Co. (Nanjing, Jiangsu, China).

### 2.13. Western Blotting

The tibias were minced and homogenized with RIPA lysis buffer and protease inhibitor cocktail (Beyotime) at 4 °C to obtain 1:9 (W/V) whole homogenate. The homogenates were centrifuged at 8000× *g* for 10 min under 4 °C to obtain the femoral proteins. Aliquots of protein extract were separated by 10% sodium dodecyl sulfate polyacrylamide gel electrophoresis (SDS-PAGE) and transferred to a polyvinylidene difluoride (PVDF) membrane, operating on a Bio-rad instrument (Hercules, CA, USA). The membrane was incubated with primary antibodies against GSK-3β (AG751, Beyotime), phospho-GSK-3β (Ser 9, AF1531, Beyotime), β–Catenin (AF0069, Beyotime), Runx2 (D1L7F, CST, Danvers, MA, USA), Nrf2 (AF7623, Beyotime), HO-1 (AF1333, Beyotime), and the loading control β–actin (2148, Abcam) or α/β–tubulin (2148S, CST). Horseradish peroxidase-conjugated secondary antibody (Beyotime) was used to enhance ECL chemiluminescence detection. The protein bands were scanned and analyzed by ImageJ software (NIH, Bethesda, MD, USA). Each protein band was normalized to its β–actin band, and the levels of phosphorylated proteins were normalized to the levels of their corresponding total proteins. The results were calculated as percentage change to the ND group.

### 2.14. Statistical Analysis

The results were analyzed using a T test or one-way analysis of variance (ANOVA) (GraphPad Prism software, 8.0 version, San Diego, CA, USA), in which the statistical significance was set at *p* < 0.05. The data were present as mean ± standard deviation (SD) of 3–8 determinations. The graphs were created by the GraphPad Prism software.

## 3. Results

### 3.1. Purification and Characterization of Osteogenic and Antioxidative Peptides from WPH

**Figure 1:**
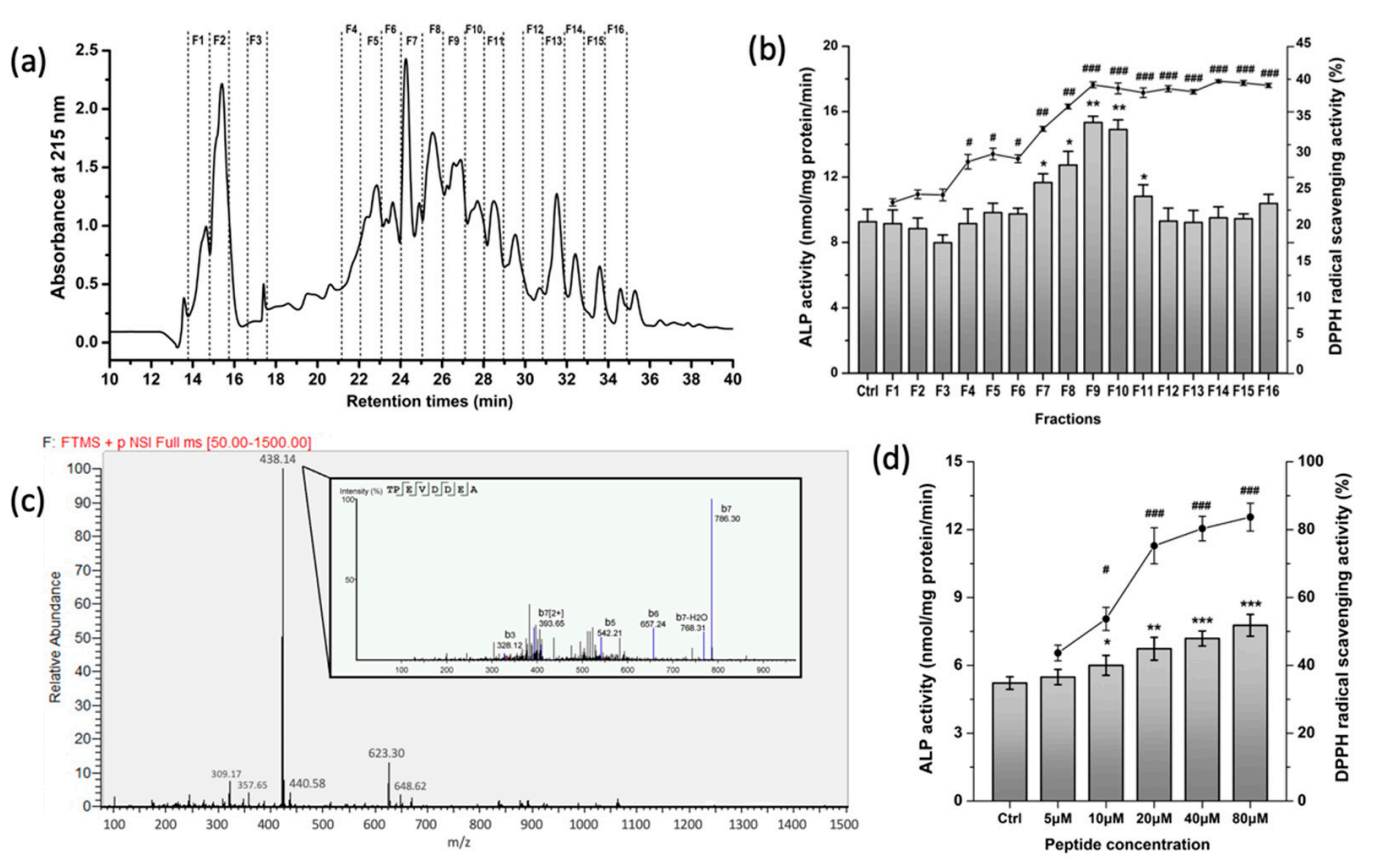
Separation and identification of the osteogenic and antioxidant peptides from WPH. (**a**) Separation of WPH on the RP-HPLC equipped with a C18 column. (**b**) Evaluation of WPH fractions on the ALP activity in MC3T3-E1 osteoblasts and the DPPH radical scavenging capacity. (**c**) Total ion chromatograms of WPH-F9 and the MS/MS spectra of the octapeptide TPEVDDEA. (**d**) Evaluation of TPEVDDEA (5–80 μM) on the ALP activity in MC3T3-E1 osteoblasts and the DPPH radical scavenging capacity. All data were shown as mean ± SD. The # (*), ## (**), and ### (***) indicated *p* < 0.05, *p* < 0.01, and *p* < 0.001, as compared to the control group, respectively.

Our previous studies reported that the WPH could regulate the homeostasis of bone metabolism in vitro and in vivo [25,27]. To further identify the specific peptides with high osteogenic and antioxidative potentials, WPH was separated into 16 fractions by RP-HPLC (Figure 1a); among which F9 possessed both significantly higher osteogenic capacity and DPPH scavenging activity than other fractions (Figure 1b). Then, the F9 was subjected to LC-MS/MS to characterize peptide sequences. A total of 16 peptides were identified (Table 2), in which the octapeptide TPEVDDEA accounted for the most abundant peptide in F9 (Figure 1c). Moreover, TPEVDDEA of 5–80 μM dose-dependently activated the ALP activity in osteoblasts and increased DPPH scavenging ratio (Figure 1d), indicating that the LPH-F9 endowed dual activities in osteogenesis and antioxidative capacity.

### 3.2. HFD-Induced Obese Mice Exhibited Bone Loss and Oxidative Damages

**Figure 2:**
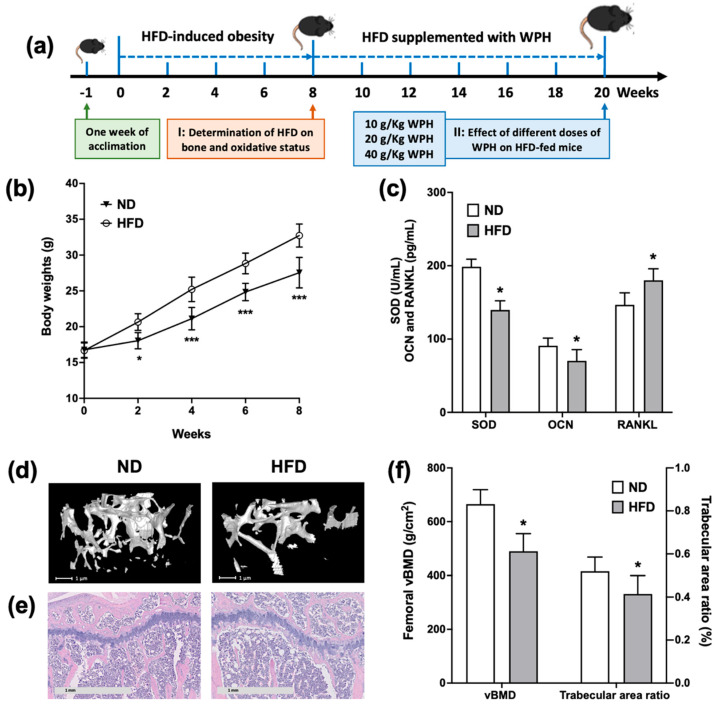
Effect of HFD feeding on the body weights, bone microstructures, and redox status of mice. (**a**) Scheme of experiment design. (**b**) Changes of body weight between ND- and HFD-fed mice. (**c**) Serum SOD, OCN, and RANKL levels. (**d**) Transverse images of distal metaphyseal femurs. (**e**) H&E-stained images of proximal tibia (5×). (**f**) Quantitation of vBMD and trabecular area ratio obtained from μCT and H&E analysis. All data were shown as mean ± SD. The * and *** indicated *p* < 0.05 and *p* < 0.001 between ND and HFD groups.

The schematic of the animal experiment was presented in Figure 2a. To investigate the effect of obesity on bone status, 5-week-old growing mice were fed with HFD for 8 weeks to induce obesity. As shown in Figure 2b, the body weights of the HFD group began to be significantly higher than those of the ND group since week 2. At week 8, HFD-fed mice exhibited significantly decreased tibial SOD activities and OCN levels, accompanied by significantly promoted tibial RANKL levels (Figure 2c). The micro-CT and histological analysis of femurs and tibias also confirmed that mice fed with HFD for 8 weeks had significantly decreased vBMD and trabecular area ratio (Figure 2d–f). The results indicated that mice fed with HFD for 8 weeks not only induced obesity but also gave rise to bone loss and imbalanced redox status.

### 3.3. WPH Restored Fat Mass and Improved Serum Lipid Levels in HFD-Fed Mice

We further evaluated the effect of WPH on ameliorating the bone loss induced by HFD in mice. As shown in Table 3, after 12 more weeks of feeding, HFD intake continuously caused excessive weight gain and fat accumulation, accompanied by abnormal glucose and lipid levels. No significant differences were observed in the final body weights and liver weights between different HFD groups, indicating WPH of different doses had no adverse effects on mice. The perirenal fat mass, as well as serum cholesterol and triglyceride levels, were significantly restored by middle and high doses of WPH, in which a high dose of WPH exhibited optimal efficiency. However, the restoration of serum glucose was insignificant in all WPH groups. Overall, WPH improved the metabolic profile of obese mice, especially on lipid levels.

### 3.4. WPH Improved Bone Minerals and Mechanical Properties in HFD-Fed Mice

Bone loss exhibits as mineral loss and reduced mechanical properties. As shown in Table 3, the femoral calcium contents of the HFD groups were significantly lower than those of the ND group. The middle and high doses of WPH groups exhibited significantly higher calcium contents than that of the HFD control group, while only a high dose of WPH significantly preserved the phosphorus contents. Moreover, the results of the bending test showed that the femurs of the HFD groups had significantly lower maximal loads and stiffness than the ND group, and only high doses of WPH significantly restored the stiffness of HFD-fed mice, indicating that high doses of WPH could reverse the brittle femurs of HFD mice.

### 3.5. WPH Repaired Bone Microstructure in HFD-Fed Mice

**Figure 3:**
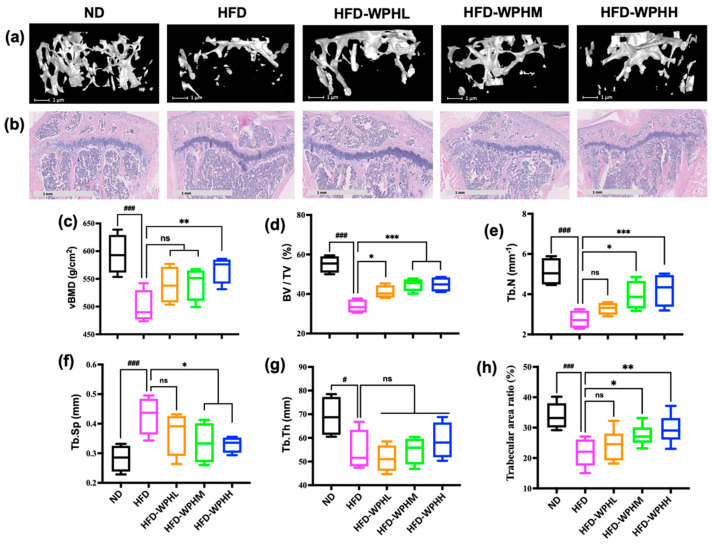
Effects of WPH on bone microstructure in HFD-fed mice. (**a**) Transverse images of distal metaphyseal femurs. (**b**) H&E staining of trabecular microstructure of proximal tibia (5×). (**c**–**h**) Quantification of bone morphometric parameters in μCT and H&E analysis. All data were shown as mean ± SD (*n* = 3). The #, and ### indicated *p* < 0.05 and *p* < 0.001, as compared to the ND group. The *, **, and *** indicated *p* < 0.05, *p* < 0.01, and *p* < 0.001, as compared to the HFD control group. The ns indicated *p* > 0.05 among groups.

The μCT analysis and H&E staining were performed to observe the bone microarchitectures. Figure 3a showed the reconstructed 3D images of trabecular bones of distal femurs, and related bone parameters were further quantified (Figure 3c–g). Compared to the dense trabeculae in ND-fed mice, mice fed with HFD had a thinner, looser, and more rod-like trabecular microarchitecture. The markedly disrupted vBMD, BV/TV, Tb.N, and Tb.Th by HFD were evidently reversed by middle and high dose of WPH; whereas none WPH groups significantly promoted the damaged Tb.Th induced by HFD. The histological analysis revealed the degraded trabeculae and increased lipid droplets within the tibial proximal metaphysis in HFD-fed mice (Figure 3b), in which the trabecular area ratio of HFD groups was significantly lower than that of ND group (Figure 3h). The WPH-supplemented HFD groups tended to have denser and more regular trabeculae arrangement, educed plate perforation and enhanced connectivity, accompanied by increased trabecular area ratios. Although the lipid droplets in cavitas medullaris had not been eliminated by WH, the amounts of lipid cavities in WPH groups were reduced. Overall, WPH possessed high potential in rescuing cancellous bone loss in HFD-fed mice.

### 3.6. WPH Readjusted Bone Remodeling in HFD-Fed Mice

**Figure 4:**
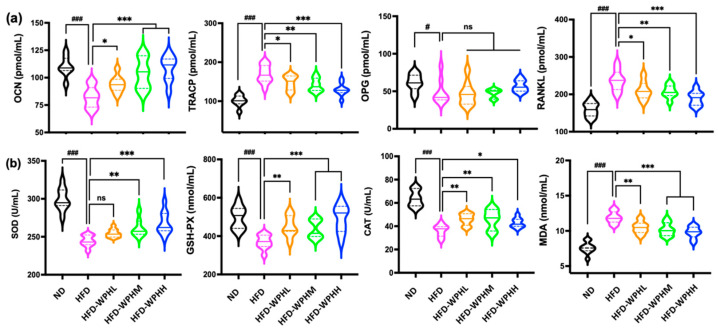
Effects of WPH on biomarkers of bone remodeling and antioxidant capacity in HFD-fed mice. (**a**) Bone remodeling biomarkers of OCN, TRACP, OPG, and RANKL in serum. (**b**) Antioxidant capacity biomarkers of SOD, GSH-PX, CAT, and MDA in serum. All data were shown as mean ± SD (*n* = 8). The #, and ### indicated *p* < 0.05 and *p* < 0.001, as compared to the ND group. The *, **, and *** indicated *p* < 0.05, *p* < 0.01, and *p* < 0.001, as compared to the HFD control group. The ns indicated *p* > 0.05 among groups.

**Figure 5:**
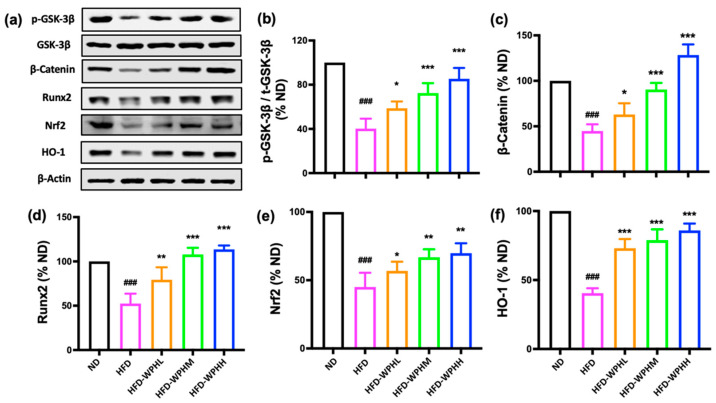
Effects of WPH on protein expressions in HFD-fed mice. (**a**–**f**) Protein expression levels of β–catenin, Nrf2, HO-1, and GSK-3β and the phosphorylated forms GSK-3β. β–Actin was used as the loading control. All data were shown as mean ± SD (*n* = 3). The ### indicated *p* < 0.001, as compared to the ND group. The *, **, and *** indicated *p* < 0.05, *p* < 0.01, and *p* < 0.001, as compared to the HFD control group.

The levels of bone formation markers (OCN and OPG) and bone resorption markers (TRACP5b and RANKL) in tibias were measured using ELISA (Figure 4a). HFD induced imbalanced bone remodeling with inhibited bone formation and activated bone turnover. The administration of WPH significantly alleviated the increased TRACP5b and RANKL levels and boosted OCN and OPG levels in HFD mice. The capacity of a high dose of WPH on regulating bone remodeling was at its utmost in different dosages. Moreover, as shown in Figure 5c, the protein expression levels of Runx2 in bone were significantly upregulated by WPH supplementation in HFD-fed mice.

### 3.7. WPH Alleviated Oxidative Stress in HFD-Fed Mice

Figure 4b showed the oxidative status of serums between different groups of mice. After 12 weeks of WPH supplementation, the increased levels of serum MDA by HFD were significantly inhibited. Meanwhile, the decreased levels of serum SOD, GSH-PX, and CAT activities in HFD-fed mice were significantly promoted by WPH of different doses. Similarly, a high dose of WPH showed superior capacity in inhibiting oxidative stress and restoring antioxidant enzymes.

### 3.8. WPH Regulated GSK-3β/Nrf2/HO-1 in HFD-Fed Mice

To further investigate the mechanism of WPH in inhibiting HFD-induced bone loss, we examined the GSK-3β/Nrf2 antioxidant response pathways, in which the GSK-3β/β–catenin axis is also fundamental for osteogenesis. As shown in Figure 5, WPH supplementation significantly inactivated the GSK-3β signaling by increasing the phosphorylation level of p-GSK-3β, which consequently promoted the expression of β–catenin. The protein levels of Nrf2, and its downstream antioxidant effector HO-1, were also upregulated by WPH in HFD-fed mice.

## 4. Discussion

It is well recognized that whey proteins are high nutritional quality proteins and there has been growing interest in developing whey-based functional foods with health-promoting effects such as on the musculoskeletal system [32]. We have summarized in a previous study that whey proteins as well as whey-derived peptides exerted efficiency on boosting osteogenesis and inhibiting bone loss [20]. We previously prepared a whey protein hydrolysate (WPH) with low-phenylalanine content, using a two-step enzymatic method and a macroporous resin column [26]. In the present study, we purified a fraction from WPH with the strongest osteogenic and antioxidant activity and further identified 16 peptides from that fraction, in which the most abundant and active peptide was the β–lactoglobulin derived octapeptide TPEVDDEA. The octapeptide of 10–80 μM not only significantly increased the activities of osteogenic marker ALP on MC3T3-E1 cells, but also exerted significant capacity on scavenging DPPH radicals. Jiang et al. (2019) purified and identified the antioxidant sequence of TPEVDDEALEKFDKALK from whey protein isolate hydrolysate, which had ABTS free radical scavenging activity [33]. Chougule et al. (2013) reported that TPEVDDEALEKFDK exhibited anti-angiogenic activity by potently inhibiting micro-vessel sprouting of human umbilical vein endothelial cells in a dose-dependent manner [34].

The long-term intake of HFD over energy consumption leads to obesity, accompanied by increased levels of ROS and decreased antioxidant defenses [35,36]. Meanwhile, the negative relationship between osteogenesis and adipogenesis within the marrow microenvironment has been described as “see-saw paradigm” [37,38]. As adipocytes and osteoblasts are both derived from the mesenchymal stem cells, the greater propensity for adipogenesis versus osteogenesis inhibits the transcription of Runx2, thus causing an increased number of adipocytes than osteoblasts. Moreover, high lipid deposition not only decreases the secretion of adiponectin but also induces high levels of reactive oxygen species (ROS) [39]. Although antioxidant enzymes constitute the defense system against ROS, the overloaded ROS will activate the production of osteoclast precursors such as macrophage colony-stimulating factor (M-CSF) and RANKL, which further triggered the key osteoclast markers tartrate-resistant acid phosphatase (TRAP); simultaneously, the increased levels of ROS aggravated the inhibition on bone formation [40,41]. Then the imbalanced bone metabolism, together with the accumulated fat adipocytes in bone tissues which affects the trabecular network integrity, led to the loss of bone minerals and the deterioration of bone microarchitecture, eventually causing osteoporosis.

In the present study, we first examined the physical and metabolic parameters of growing mice fed with HFD for 8 weeks. The HFD-induced obese mice exerted significantly increased body weight, accompanied by deteriorated bone architecture, as well as disturbed redox homeostasis and bone metabolism. In light of these premises, we further explored the administration of low, middle, and high doses of WPH for 12 weeks on ameliorating the bone loss and oxidative damage induced by HFD. Our results showed that the supplementation of 2% and 4% WPH ameliorated bone loss via regulating oxidative stress and activating the GSK-3β/Nrf2/HO-1 signaling pathway.

Increasing evidence indicated that the activation of Nrf2 can resist oxidative stress. At the level of ROS increased, the bounded NRF2 and Kelch-like ECH-associated protein 1 (KEAP1) begin to dissociate, which allows the translocation of Nrf2 into the nucleus and activates the expression of downstream antioxidant proteins such as HO-1, Gclc, and NQO1, which, in turn, quenches ROS [42,43]. Our in vivo research showed that HFD-fed mice exerted ROS levels that aggravated the loss of bone mass and deterioration of bone structure; while the supplementation of WPH ameliorated HFD-induced bone loss via regulating OPG/RANKL axis and GSK-3β/Nrf2/HO-1 signaling pathway, in which Nrf2 might act as a central role of the antiosteoporotic effect of WPH. Chen et al. (2021) researched that Nrf2 gene knockout ovariectomized mice displayed a significantly reduced bone mass than the control mice, illustrating Nrf2 was the critical factor for antiosteoporosis [44]. Therefore, further study could focus on the interaction between WPH and Nrf2.

In addition, the activation of Wnt/GSK-3β signaling pathways could also regulate Nrf2 activation and facilitate its accumulation in the nucleus for the promotion of HO-1 gene expression [10,45]. As for the Wnt/GSK-3β signaling pathway, the canonical β–catenin-dependent pathway is vital for osteogenesis, in which the activated Wnt proteins inhibit the activity of GSK-3β and thereby accumulate the hypophosphorylation of β–catenin [46], which further trigger the gene expression of Runx2. Sun et al. (2022) reported that hops extract and its active component xanthohumol ameliorated bone loss induced by iron overload via activating Akt/GSK3β/Nrf2 pathway [47], indicating the direction on identifying the inner molecular mechanism of WPH on activating Akt signaling pathway.

Nowadays, the global obesity epidemic has put forward the demand for better nutritional intervention strategies, especially the exploitation of food-derived compounds. Whey proteins and their derived bioactive peptides have been well recognized as high nutritional quality protein/peptide resources. Thus, this study, for the first time, provided evidence for the mechanism by which whey protein hydrolysates ameliorated HFD-induced bone loss by regulating OPG/RANKL axis and GSK-3β/Nrf2/HO-1 signaling pathway; and giving a potential role for developing whey-based functional foods with bone-promoting health benefits.

## Figures and Tables

**Table 1:** Composition of the diets.

Ingredient (g/kg)	ND	HFD	HFD-WPHL	HFD-WPHM	HFD-WPHH
Casein	200	200	190	180	160
WPH ^†^	/	/	12	24	48
Lard oil	20	177.5	177.5	177.5	177.5
l–Cysteine	3	3	3	3	3
Corn starch	452.2	72.8	72.8	72.8	72.8
Maltodextrin	75	100	100	100	100
Sucrose	172.8	172.8	172.8	172.8	172.8
Cellulose ^$^	50	50	48	46	42
Corn oil	25	25	25	25	25
Mineral mixture	45	45	45	45	45
Vitamin mixture	10	10	10	10	10
Choline	2	2	2	2	2
Ratio of fat/total calorie (%)	15	45	45	45	45

^†^ HFD-WPH diets were prepared by using the replacement of casein with WPH, in which the accurate weights were calculated according to the protein content of casein (~95 g/100 g) and WPH (~75 g/100 g). ^$^ The exceeded weight of WPH replaced equal amount of cellulose.

**Table 2:** The information of the identified peptides of WPH-F9.

No.	Peptide Sequence	*m*/*z*	MW ^#^	Source (Protein Accession)
1	SSRQP	287.65	573.29	Glycosylation-dependent cell adhesion molecule 1 (P80195)
2	LNENK	309.17	616.318	Beta-lactoglobulin (P02754)
3	TPKAKDKNKH	389.56	1165.65	Osteopontin (P31096)
4	TPEVDDEALEK	415.19	1245.58	Beta-lactoglobulin (P02754)
5	TEAQEDGQSTSE	427.17	1281.50	Endoplasmin (Q95M18)
6	TPEVDDEA	438.18	874.36	Beta-lactoglobulin (P02754)
7	VPYPQRDMPIQ	447.89	1343.68	Beta-casein
8	TPKAKDKNKHSN	456.58	1368.73	Osteopontin (A0A077KSH2)
9	VSNAEGSQPDDSSS	459.85	1379.56	Perilipin (A1L5C2)
10	YLYEIAR	464.25	926.49	Albumin (P02769)
11	ENSAEPEQS	495.70	989.39	Beta-lactoglobulin (P02754)
12	NKPEDETH	485.22	968.42	Glycosylation-dependent cell adhesion molecule 1 (P80195)
13	KVPQVSTPTLVEVSR	547.32	1638.93	Albumin (P02769)
14	RNAVPITPTLN	598.34	1194.67	Alpha-S2-casein (P02663)
15	QSEEQQQTEDE	675.26	1350.53	Beta-casein (A0A452DHW7)
16	TEAQEDGQSTSE	427.17	1281.50	Endoplasmin (Q95M18)

^#^ MW, molecular weight, Da.

**Table 3:** Effect of WPH on body and tissue weights, metabolic profile, femoral composition, and femoral mechanical properties in HFD-fed mice^*^.

	ND	HFD	HFD-WPHL	HFD-WPHM	HFD-WPHH
Body and tissue weight (g)					
Final body weight	36.63 ± 3.25 ^a^	50.52 ± 3.24 ^b^	49.74 ± 4.02 ^b^	48.49 ± 3.37 ^b^	48.16 ± 3.76 ^b^
Liver	1.13 ± 0.11 ^a^	1.67 ± 0.20 ^b^	1.60 ± 0.18 ^b^	1.58 ± 0.21 ^b^	1.56 ± 0.22 ^b^
Epididymis fat	0.25 ± 0.05 ^a^	0.72 ± 0.13 ^b^	0.73 ± 0.14 ^b^	0.70 ± 0.06 ^b^	0.68 ± 0.10 ^b^
Mesenteric fat	0.11 ± 0.05 ^a^	0.84 ± 0.10 ^b^	0.80 ± 0.12 ^b^	0.82 ± 0.09 ^b^	0.81 ± 0.10 ^b^
Perirenal fat	0.40 ± 0.06 ^a^	1.57 ± 0.08 ^c^	1.55 ± 0.06 ^c^	1.44 ± 0.07 ^b^	1.38 ± 0.09 ^b^
Metabolic profile					
Cholesterol (nmol/mL)	3.28 ± 0.10 ^a^	7.64 ± 0.13 ^d^	7.33 ± 0.12 ^d^	6.27 ± 0.13 ^c^	5.83 ± 0.13 ^b^
Triglyceride (nmol/mL)	1.01 ± 0.03 ^a^	1.80 ± 0.08 ^d^	1.75 ± 0.04 ^d^	1.46 ± 0.11 ^c^	1.28 ± 0.04 ^b^
Glucose (mmol/L)	0.78 ± 0.05 ^a^	1.45 ± 0.08 ^b^	1.43 ± 0.06 ^b^	1.40 ± 0.05 ^b^	1.41 ± 0.06 ^b^
Femoral composition and mechanical properties					
Femoral calcium (mg/g)	65.18 ± 3.14 ^c^	47.33 ± 2.28 ^a^	49.40 ± 2.09 ^a^	53.29 ± 2.51 ^b^	55.47 ± 2.67 ^b^
Femoral phosphorus (mg/g)	134.01 ± 9.84 ^c^	108.66 ± 5.37 ^a^	110.43 ± 6.52 ^ab^	112.41 ± 4.28 ^b^	116.54 ± 6.33 ^b^
Femoral maximal load (N)	46.76 ± 2.07 ^b^	35.28 ± 4.31 ^a^	35.54 ± 3.58 ^a^	36.50 ± 3.63 ^a^	35.33 ± 3.75 ^a^
Femoral stiffness (N)	102.54 ± 8.86 ^c^	79.53 ± 5.26 ^a^	82.23 ± 5.04 ^ab^	85.34 ± 4.67 ^ab^	88.23 ± 5.30 ^b^

^*^ Different lowercases in the same line were significantly different at *p* < 0.05.

## Data Availability

Not applicable.

## Abbreviations

alkaline phosphatase, ALP; bone mineral density, BMD; catalase, CAT; 1,1-diphenyl-2-picrylhydrazyl, DPPH; enzyme-linked immunosorbent assay, ELISA; glutathione peroxidase, GSH-Px; glycogen synthase kinase-3β, GSK-3β; heme oxygenase 1, HO-1; interleukin 6, IL-6; mitogen-activated protein kinase, MAPK; molecular weight, MW; nuclear factor κB, NF–κB; osteocalcin, OCN; osteoprotegerin, OPG; phenylalanine, Phe; superoxide dismutase, SOD; receptor activator of nuclear factor kappa B, RANK; receptor activator of nuclear factor kappa B ligand, RANKL; reactive oxygen species, ROS; runt-related transcription factor 2, Runx2; tumor necrosis factor α, TNF–α; tartrate-resistant acid phosphatase 5b, TRACP5b, whey protein concentrate, WPC; whey protein hydrolysate, WPH.
